# Osteochondral regeneration of the femoral medial condyle by using a scaffold-free 3D construct of synovial membrane-derived mesenchymal stem cells in horses

**DOI:** 10.1186/s12917-021-03126-y

**Published:** 2022-01-22

**Authors:** Daiki Murata, Shingo Ishikawa, Takafumi Sunaga, Yasuo Saito, Takeshi Sogawa, Koichi Nakayama, Seiji Hobo, Takashi Hatazoe

**Affiliations:** 1 Department of Veterinary Clinical Science, Joint Faculty of Veterinary Medicine, Kagoshima University, Kagoshima, Japan; 2 Department of Regenerative Medicine and Biomedical Engineering, Center for Regenerative Medicine Research, Faculty of Medicine, Saga University, Saga, Japan; 3 Division of Veterinary Science, Graduate School of Life and Environmental Biosciences, Osaka Prefecture University, Izumi-Sano, Japan; 4 Veterinary Teaching Hospital, Faculty of Veterinary Medicine, Kagoshima University, Kagoshima, Japan; 5 Department of Veterinary Clinical Sciences, Graduate School of Veterinary Medicine, Hokkaido University, Sapporo, Japan

**Keywords:** Horse, Mesenchymal stem cell, Regeneration, Subchondral bone cyst, Synovial membrane

## Abstract

**Background:**

Medical interventions for subchondral bone cysts in horses have been extensively studied. This study investigated the regeneration of articular cartilage and subchondral bone with scaffold-free three-dimensional (3D) constructs of equine synovial membrane-derived mesenchymal stem cells (SM-MSCs) isolated from three ponies and expanded until over 1.0 × 10^7^ cells at passage 2 (P2).

**Results:**

SM-MSCs were strongly positive for CD11a/CD18, CD44, and major histocompatibility complex (MHC) class I; moderately positive for CD90, CD105, and MHC class II; and negative for CD34 and CD45 on flow cytometry and differentiated into osteogenic, chondrogenic, and adipogenic lineages in the tri-lineage differentiation assay. After culturing SM-MSCs until P3, we prepared a construct (diameter, 6.3 mm; height, 5.0 mm) comprising approximately 1920 spheroids containing 3.0 × 10^4^ cells each. This construct was confirmed to be positive for type I collagen and negative for type II collagen, Alcian blue, and Safranin-O upon histological analysis and was subsequently implanted into an osteochondral defect (diameter, 6.8 mm; depth, 5.0 mm) at the right femoral medial condyle. The contralateral (left femoral) defect served as the control. At 3 and 6 months after surgery, the radiolucent volume (RV, mm^3^) of the defects was calculated based on multiplanar reconstruction of computed tomography (CT) images. Magnetic resonance (MR) images were evaluated using a modified two-dimensional MR observation of cartilage repair tissue (MOCART) grading system, while macroscopic (gross) and microscopic histological characteristics were scored according to the International Cartilage Repair Society (ICRS) scale. Compared to the control sites, the implanted defects showed lower RV percentages, better total MOCART scores, higher average gross scores, and higher average histological scores.

**Conclusions:**

Implantation of a scaffold-free 3D-construct of SM-MSCs into an osteochondral defect could regenerate the original structure of the cartilage and subchondral bone over 6 months post-surgery in horses, indicating the potential of this technique in treating equine subchondral bone cysts.

## Background

Of all joint diseases in animals, intra-articular fractures, osteochondrosis, and subchondral bone cysts of thoroughbred racehorses are serious diseases that cause rapid progression of osteoarthritis (OA) at a young age. Progression of OA can cause subchondral bone sclerosis in addition to articular cartilage degeneration, necessitating simultaneous interventions for the bone and cartilage [1]. Because equine OA progresses owing to repeated load on the joints during races, the lesions of cartilage degeneration and bone sclerosis are confined to the joint loading site [2]. Therefore, the mosaic-plasty method, wherein healthy bone and cartilage at a joint non-loading site are collected and autografted to a joint-loading site, has been established [3]. Although this method can improve symptoms, it is associated with problems such as the damage to healthy osteocartilage at the non-loading site and limitations in the amount of transplantable osteocartilage [4]. Transplantation of chondrocytes seeded on artificial bone such as hydroxyapatite and artificial substrates such as atelocollagen has been studied, but this technique cannot regenerate hyaline cartilage [5]. Moreover, because artificial bone has poor bio-absorbability and is retained in the body for a long time, it supports the formation of biofilms during infection. For procedures involving the implantation of artificial substrates, low-immunogenicity materials such as collagen have been used, but absorption and substitution of these materials into a self-matrix is necessary. Unlike bone, in which multiple cells coordinate degradation and matrix renewal, cartilage metabolism is so slow that it is carried only by chondrocytes. Therefore, when artificial collagen material is transplanted, a system for excluding the artificial material will persist in the new cartilage for a long period of time and may hinder the formation of new hyaline cartilage. Other studies have pointed out additional problems, such as the limited number of chondrocytes collected from cartilage tissue [6] and the dedifferentiation of chondrocytes during the culturing process [7]. Considering these findings, a method of transplanting mesenchymal stem cells (MSCs) without using an artificial substrate would be suitable for regenerating hyaline cartilage. The authors had previously proposed a three-dimensional (3D) construct wherein stem-cell aggregates are three-dimensionally arranged in a mould without using any substrate such as an artificial scaffold and the 3D construct thus obtained is transplanted into osteochondral defects to simultaneously regenerate bone and cartilage in animal experiments [8, 9].

Recent research on regenerative medicine has yielded a method of sheeting or gelling with stem cells [10, 11]. However, by focusing on the characteristic of cells to aggregate and the property of aggregates to fuse with adjacent aggregates, we set up a new cell-therapy strategy in which 3D constructs were created with stem cells and implanted into osteochondral defects to regenerate bone and cartilage. Specifically, we created 3D constructs made of autologous MSCs and implanted them into osteochondral defects at a non-loading site (the femoral trochlear groove) [8, 9] and a loading site (the femoral medial condyle) [12]. Thereafter, we proved osteochondral regeneration within 6 months after implantation by performing histopathological evaluation [8, 9], computed tomography (CT) [8], and magnetic resonance (MR) imaging [12]. In addition, we succeeded in separating and culturing MSCs from the synovial fluid (SF) of thoroughbred racehorses that developed a joint disease [13]. In contrast, MSCs can hardly be separated from the SF in a healthy joint [13]. We also found that the characteristics of SF-derived MSCs (SF-MSCs) were similar to those of synovial membrane (SM)-derived MSCs (SM-MSCs); we reported that SF-MSCs and SM-MSCs are superior to equine bone marrow-derived MSCs and equine adipose tissue-derived MSCs in cartilage differentiation ability, with SF-MSCs differentiating into chondrocytes and forming a sheet-like structure strongly stained by Alcian Blue (which we named the “chondro-sheet”) [13]. Therefore, we decided to study the possibility of achieving osteochondral regeneration at the femoral medial condyle of horses by using 3D constructs composed of autologous SM-MSCs and not SF-MSCs as they are difficult to separate from normal joints.

## Methods

### Animals

Three ponies denoted equine Nos. 1 to 3 were purchased from an farmer (owner) and then used in this study. They were all 3 years old, and their body weights were approximately 140 kg (No. 1), 160 kg (No. 2), and 200 kg (No. 3). All procedures in this study were approved by the Animal Care and Use Committee of Kagoshima University (approval no. VM16015).

### Isolation and expansion of SM-MSCs

The surgery to collect the SM was performed under general anaesthesia using oxygen and isoflurane inhalation following pre-medication with sedatives such as medetomidine (Domitor, Zenoaq; 7 μg/kg intravenous injection) and midazolam (Dormicum, Astellas Pharma; 30 μg/kg intravenous injection) and analgesics such as ketamine (Ketalar, Daiichi Sankyo Propherma; 1 mg/kg intravenous injection). The ponies were laid on their back with their carpus bent to 125 degrees. The right radiocarpal joint was incised on the medial side of the tendon of the extensor carpi radialis and between the tendon of the extensor carpi radialis and the tendon of the extensor digitorum communis; thereafter, approximately 250 mg of SM tissue per animal was aseptically obtained using an arthroscope. The tissues were minced and digested for 90 min in phosphate-buffered saline (PBS) containing 0.1% collagenase (Collagenase type I, Worthington Biochemical). The digested cell suspensions were filtered through a 70-μm pore diameter membrane (Cell Strainer, BD) and centrifuged at 160×*g* for 5 min at room temperature. After decanting the supernatant, the pellet was rinsed with PBS and centrifuged. After removing the supernatant, the pellet was resuspended and plated in a 75-cm^2^ culture flask (TCF-75 N-V; Wuxi NEST Biotechnology) in primary culture medium (PCM):Dulbecco’s modified Eagle medium (DMEM, Life Technologies) containing 10% foetal bovine serum (FBS; HyClone FBS, Thermo Scientific) and 1% antibiotic-antifungal preparation (100 U/mL Penicillin G, 100 μg/mL streptomycin, and 0.25 μg/mL amphotericin B; Antibiotic-Antimycotic, Life Technologies). After incubation at 37 °C under 5% CO_2_ for 3 days, cells adhering to the bottom of the dish were washed with PBS and cultured in PCM. Cells were harvested with a recombinant enzyme (TrypLE Select, Life Technologies) for dissociation and centrifuged on day 6 in Passage 0 (P0). After decanting the supernatant, the pellet was rinsed with secondary culture medium (SCM):xeno-free and serum-free MSC culture medium (MSC NutriStem XF Basal Medium, Biological Industries USA) containing supplements (MSC NutriStem XF Supplement Mix, Biological Industries USA), reduced serum (2%) MSC culture medium (MesenPro RS Medium, Life Technologies), and 1% antibiotic-antifungal preparation (Antibiotic-Antimycotic, Life Technologies); cells were seeded at 2.0 × 10^6^ cells per 225-cm^2^ culture flask (CellBIND Surface Cell Culture Flasks, Corning) and cultured until 80% confluence. This serial process of passage was repeated until the number of the cells reached 1.0 × 10^7^. The number of cells was measured at every passage by using a cell counter (TC10, BioRad) to determine the cell proliferation rate. The proliferation rates were determined in terms of the cell doubling number, cell doubling time, and daily duplication rate using the following formulas:

$$\mathrm{Cell}\ \mathrm{doubling}\ \mathrm{number}=\ln \left(\mathrm{final}\ \mathrm{number}\ \mathrm{of}\ \mathrm{cells}/\mathrm{initial}\ \mathrm{number}\ \mathrm{of}\ \mathrm{cells}\right)/\ln (2)$$

$$\mathrm{Cell}\ \mathrm{doubling}\ \mathrm{time}\ \left[\mathrm{days}\right]=\mathrm{Cell}\ \mathrm{culture}\ \mathrm{time}/\mathrm{cell}\ \mathrm{doubling}\ \mathrm{number}$$

Immunological surface markers and multipotency of the cells were analysed and constructs were created at P3.

### Flow cytometry for immunological surface markers of SM-MSCs

Ten-thousand cells were resuspended in 500 μL of staining buffer (SB) (PBS containing 1% FBS) and incubated for 30 min at 4 °C with 20 μg/mL antibodies (mouse immunoglobulin G) against cluster of differentiation (CD)11a/CD18 (gifted), CD34 (BD), CD44 (AbD Serotec), CD45 (BD), CD90 (BD), CD105 (AbD Serotec), MHC class I (gifted), and MHC class II (gifted), as previously reported [13–16]. After incubation, the antibodies against CD11a/CD18, CD44, MHC class I, and MHC class II were coupled with secondary got antibodies conjugated to fluorescein isothiocyanate (FITC; FITC anti-mouse IgG, Rockland lmmunologicals). The FITC-labelled cells were washed with PBS and resuspended in 500 μL of SB for fluorescence-activated cell sorter analysis. Cell fluorescence was evaluated as a strong shift in the mean fluorescence intensity (MFI) on the flow cytometry instrument (FACS Calibur, BD). A non-specific antibody (Non-specific FITC mouse immunoglobulin G1κ, BD) was used as the negative control.

### Induction and evaluation for tri-lineage differentiation of SM-MSCs

For osteogenic differentiation, SM-MSCs were plated onto 6-well plates (6 Well Plate-N, Wuxi NEST Biotechnology) on a basal medium at a starting approximate density of 3000 cells/cm^2^. After 24 h of incubation, the basal medium was replaced with a special medium for osteogenesis (Osteogenic Differentiation Medium, Lonza), which included the basal medium and was supplemented with 100 μM ascorbic acid, 10 mM β-glycerophosphate, and 1 μM dexamethasone. The special medium was replaced every 3 days for 2 weeks. Two weeks after the osteogenic induction, the production of calcium apatite crystals in the extracellular matrix was evaluated by staining culture plates with alizarin red protocol, indicating the presence of calcium apatite crystals.

Chondrogenic differentiation was induced in the plate culture for 2 weeks. Thirty-thousand cells were resuspended into each well of a 6-well plate with 2 mL of chondrogenic induction medium (Chondrogenic Differentiation Medium, Lonza), which was composed of basal medium and supplements, including 4.5 g/L d-glucose, 350 μM l-proline, and 100 nM dexamethasone. The special medium was replaced once every 3 days for 2 weeks. Two weeks after induction, production of mucopolysaccharide in the extracellular matrix was determined by staining the plate with Alcian blue. The differentiated cells were fixed onto the culture plate by methanol, and treated with 3% acetic acid. The plates onto which the cells adhered were stained with Alcian blue (pH 2.5) for 90 min.

Adipogenic differentiation was induced when the cells reached a density of 20,000 cells/cm^2^ on 6-well plates in SCM. After pre-incubation for 24 h, SCM was replaced with adipogenic induction medium (Osteogenic Differentiation Medium, Lonza) composed of basal medium supplemented with 4.5 g/L D-glucose, 100 μM indomethacin, 10 μg/mL insulin, 0.5 mM 3-isobutyl-1-methylxanthine, 1 μM dexamethasone, and 10% rabbit serum. Three days later, adipocyte-specific intracellular lipids were stained with oil red O.

### Creation of a 3D construct of SM-MSCs

**Fig. 1:**
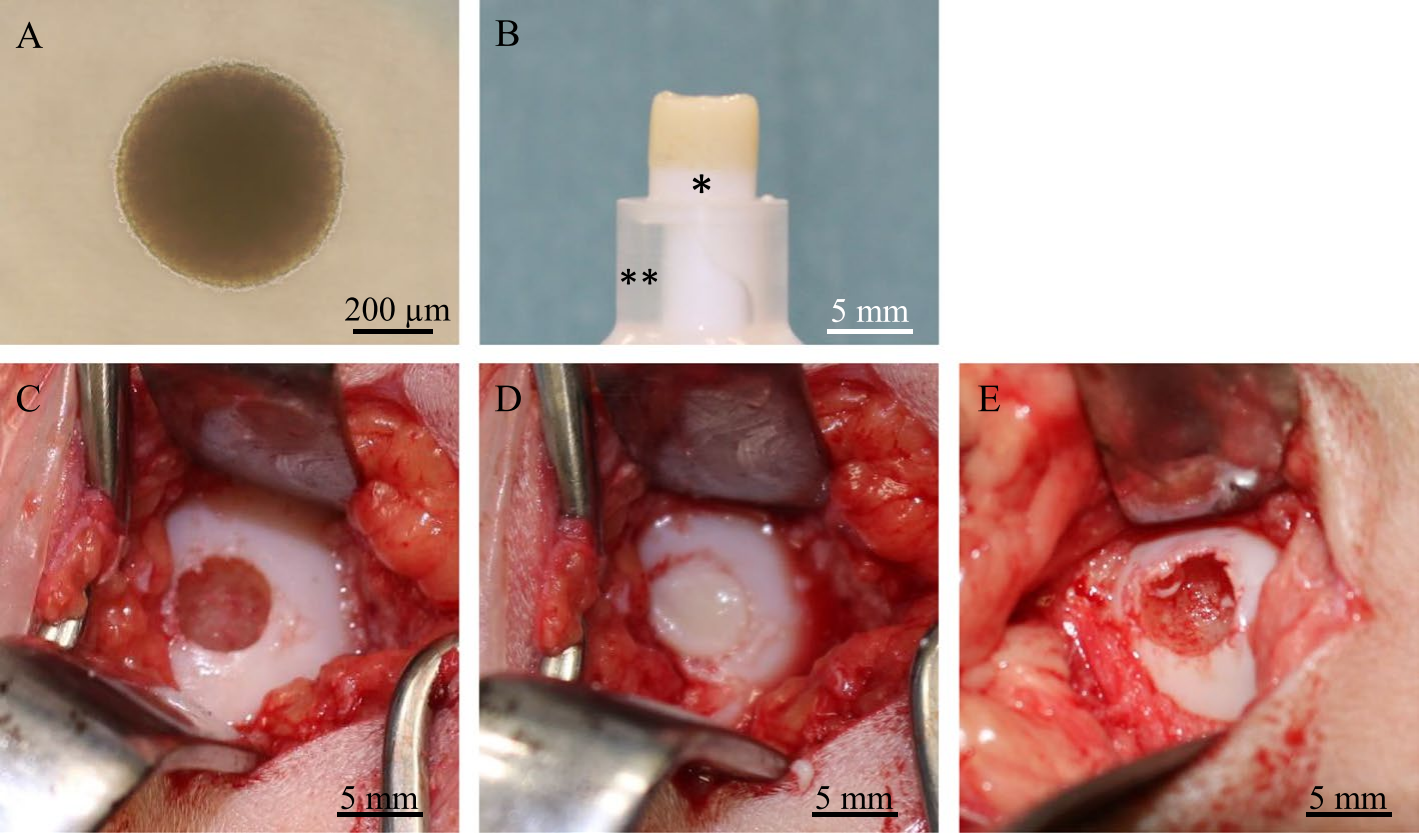
Three-dimensional construct preparation and surgical procedure. The synovial membrane-derived mesenchymal stem cells formed a spheroid with a diameter of approximately 600 μm (**A**). A columnar construct (6.3 mm in diameter and 5 mm in height) was produced for implantation (**B**). A cylindrical osteochondral defect was created in each medial condyle before implantation (**C**). The construct was autografted into the osteochondral defect in the right hind limb (**D**). Nothing was implanted into the left limbs (control defects) (**E**). The white portion under the construct is a construct sill (asterisk) and the clear portion under the sill is a mould (double asterisks)

At least 5.0 × 10^7^ SM-MSCs were used to produce an autologous construct. The cells were inoculated into twenty 96-well plates (PrimeSurface MS-9096 U, Sumitomo bakelite) with 2.5 × 10^4^ cells per well. After the plates were incubated undisrupted for 48 h, the cells formed a spheroid with a diameter of approximately 600 μm in the bottom of the well (Fig. 1a). Approximately 1920 such spheroids were placed into a cylindrical mould and incubated in SCM until implantation (7 days). When the mould was carefully removed, a columnar construct (6.3 mm in diameter and 5 mm in height) appeared and was used for the subsequent autologous implantation (Fig. 1b). The general outline of this method of construction has been previously reported [8, 9].

### Implantation of the 3D construct

The implant surgery was performed under general anaesthesia using oxygen and isoflurane inhalation following pre-medication with sedatives (midazolam, 30 μg/kg intravenous injection; alfaxalone [Alfaxan, Meiji Seika], 2 mg/kg intravenous injection) and analgesics (butorphanol [Vetorphale, Meiji Seika], 100 μg/kg intravenous injection). The ponies were laid on their back with their knees bent to 90 degrees. Both femorotibial joints were incised from the outside between the medial and intermediate patellar ligaments, and the femoral medial condyle was exteriorized. Using a drill with an outer diameter of 6.5 mm, a cylindrical osteochondral defect to a depth of 5 mm was created in each femoral medial condyle. The columnar construct (6.3 mm in diameter and 5 mm in height) composed of SM-MSC spheroids was autografted into the osteochondral defect in the right hindlimb (Fig. 1c-d), and nothing was implanted in the left hindlimb (control defect, Fig. 1e). The gap between the defect (diameter, 6.5 mm) and the construct (diameter, 6.3 mm) was filled up by a press fit after implantation.

### Histological assessment of the 3D construct

Seven days after fabrication, a construct was fixed in 10% NBF for 1 week and was paraffin-embedded after longitudinal sectioning in the central part of the construct. Serial sections (3-μm thick) were placed on glass slides and evaluated with haematoxylin & eosin (HE) staining, Masson’s trichrome staining, Safranin O staining, Alcian blue staining, and immunohistochemistry using specific antibodies against collagen type I (1310–01, 1:100 dilution; Southern Biotechnology Associates) and type II (1320–01, 1:100 dilution; Southern Biotechnology Associates) and an Avidin-Biotin Enzyme Complex system (VECTASTAIN ABC Standard Kit, Vector Laboratories).

### CT assessment of the osteochondral defects

The implants and the osteochondral defects in both stifles were followed up every 3 months for 6 months (0, 3, and 6 months) after surgery by using a CT scanner (Aquilion, TOSHIBA). The animals were sedated by medetomidine and midazolam, as described above, and retained in the dorsal position. Both of their stifles were examined by taking a series of CT scans and analysed using multi-planar reconstruction imaging to reconstruct two-dimensional (2D) and 3D images of the interior surfaces of the bone defects. Longitudinal section images were obtained in the lateral views of the cylindrical defect with 0.5-mm slice thickness. The radiolucent area of the defect in each 2D sectional image was measured, and these areas were integrally calculated to determine the radiolucent volume (RV) of the defect.

### MR assessment of the osteochondral defects

The implants and the osteochondral defects in both stifles were observed 6 months after surgery by using an MR 3.0 Tesla imager (ECHELON OVAL, HITACHI). After arthroscopic observation, all three ponies were euthanized by exsanguination after overdosed sedative (sodium thiopental [Labonal, Mitsubishi Tanabe], 12 mg / kg intravenous injection) and muscle relaxant (succinylcholine [Suxamethonium, Astellas Pharma], 1 mg / kg intravenous injection) under general anaesthesia using oxygen and isoflurane inhalation. And then both knees were set up in the coil for a human head without arthrotomy. The findings of MR images were also scored using a modified 2D MR observation of cartilage repair tissue (MOCART) grading scale.

### Arthroscopic (macroscopic) assessment of the osteochondral defects

Macroscopic findings of the three ponies were obtained arthroscopically and scored 6 months after surgery by using the International Cartilage Repair Society (ICRS) gross grading scale.

### Histological assessment of the osteochondral defects

After MR observation, both distal femurs were fixed in 10% NBF for 2 weeks and were longitudinally sectioned parallel to the trochlear groove. The tissue was paraffin-embedded following decalcification with formic acid for 1 week. Serial sections (6-μm thick) were placed on glass slides and evaluated with HE staining, Masson’s trichrome staining, Safranin O staining, and immunohistochemistry using specific antibodies against collagen type I (1:00 dilution, Southern Biotechnology Associates) and type II (1:100 dilution, Southern Biotechnology Associates) and an Avidin-Biotin Enzyme Complex system (VECTASTAIN ABC Standard Kit, Vector Laboratories). The histopathological findings were also scored using the ICRS histological grading scale.

### Statistical analysis

All numeric data are reported as means ± standard error (SE). RVs, 2D-MOCART scores, and ICRS scores were compared between the implanted and control groups with the Mann-Whitney U test and *p* < 0.05 was considered statistically significant.

## Results

### Proliferation and immunological surface markers of SM-MSCs

**Fig. 2:**
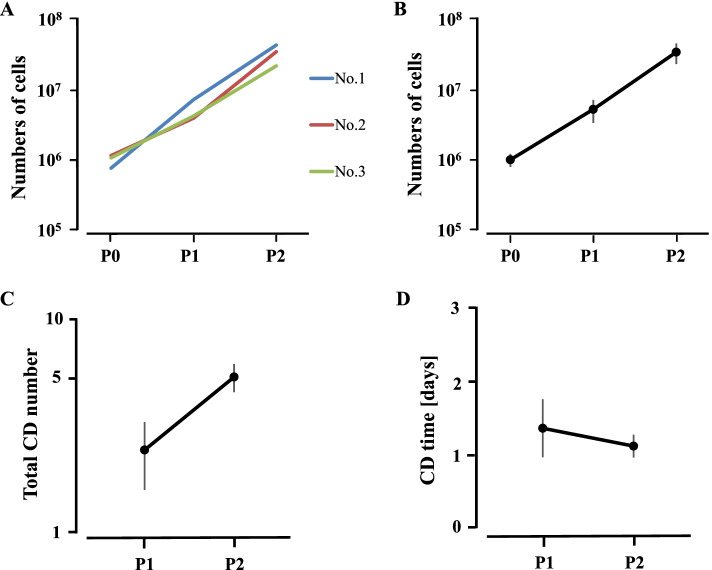
Proliferation of synovial membrane-derived mesenchymal stem cells (SM-MSCs) collected from three ponies. Growth curves (**A** and **B**) up to 1.0 × 10^7^, Cell doubling numbers (**C**), and cell doubling times (**D**) from passages 0 to 2 (P0–P2) of SM-MSCs. Cell doubling number = ln(Nf/Ni)/ln(2). Cell doubling time = cell culture time / cell doubling number. Nf, final number of cells; Ni, initial number of cells

**Fig. 3:**
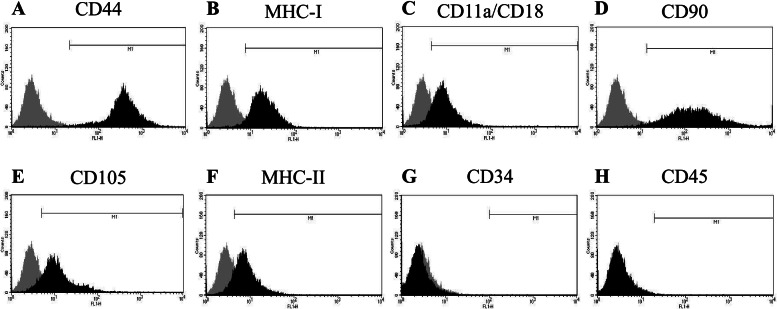
Results of flow cytometry using immunological markers on synovial membrane-derived mesenchymal stem cells. The grey area represents the negative control. The horizontal line in individual histograms indicates the population of the positive cells

SM-MSCs adhering to the bottom of the culture flask were spindle-shaped and sufficiently proliferative to form over 1.0 × 10^7^ cells by P2 (Fig. 2a-b). Cell-doubling numbers were 2.36 ± 0.81 and 5.08 ± 0.75 at P1 and P2, respectively (Fig. 2c), while the corresponding cell-doubling times were 1.36 ± 0.4 and 1.12 ± 0.16 days, respectively (Fig. 2d). Flow cytometry for assessment of immunological markers on the SM-MSCs showed that the cells presented evidently positive shifts in MFI with antibodies against CD44 and MHC class I (Fig. 3a-b). On the other hand, the positive signals against CD11a/CD18, CD90, CD105, and MHC class II were not so strong (Fig. 3c-f), and no signal was detected with antibodies against CD34 and CD45 (Fig. 3g-h). The percentages of cells positive for these molecular markers are presented in Table 1. Numerical values are shown as mean ± standard deviation.

**Table 1:** Percentages (%) of cells positive for specific molecular markers by flow cytometry

Molecular markers	CD11a/ CD18	CD34	CD44	CD45	CD90	CD105	MHC-I	MHC-II
Positive cells (%)	90.2 ± 2.4	0.4 ± 0.4	98.5 ± 1.5	0.4 ± 0.1	84.3 ± 13.0	75.1 ± 12.2	95.2 ± 0.7	88.3 ± 2.0

MHC, major histocompatibility complex

### Tri-lineage differentiation of SM-MSCs

**Fig. 4:**
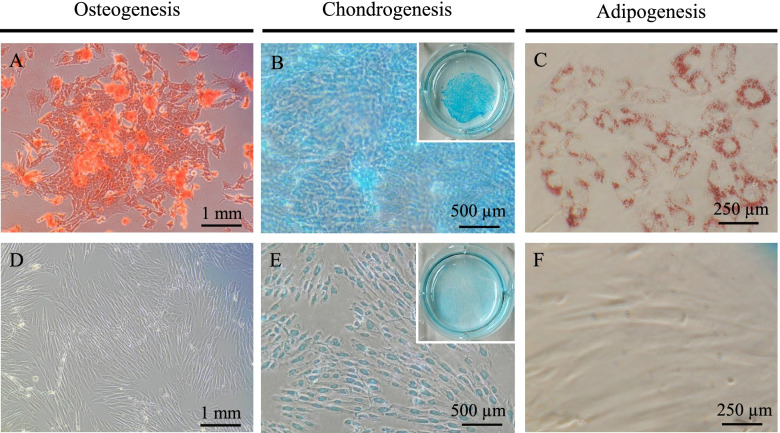
Results of tri-linage differentiation. Synovial membrane-derived mesenchymal stem cells (SM-MSCs) were cultured under osteogenic induction and stained with alizarin red. Scale bar = 1 mm (**A**). SM-MSCs induced to chondrocytes were stained with Alcian blue. Scale bar = 500 μm (**B**). Adipogenic induction of SM-MSCs was evaluated by staining with oil red O. Scale bar = 250 μm (**C**). The negative controls were cultured with incomplete culture medium during the corresponding periods of time taken to induce osteogenic (**D**), chondrogenic (**E**), and adipogenic differentiation (F)

After osteogenic induction, SM-MSCs became circular in shape and produced a specific calcium apatite crystal matrix, which was stained with Alizarin red (Fig. 4a). After the chondrogenic culture in plate, SM-MSCs were transformed to a stone-like cell shape and generated a gelatinous monolayer sheet that was intensely stained with Alcian blue (Fig. 4b). Adipogenic induction of SM-MSCs resulted in adipocyte-like flattened cells with small lipid vesicles that stained positively with oil red O (Fig. 4c). Negative controls for osteogenic, chondrogenic, and adipogenic induction are shown in Figs. 4d, e, and f, respectively.

### Characteristics of the 3D construct

**Fig. 5:**
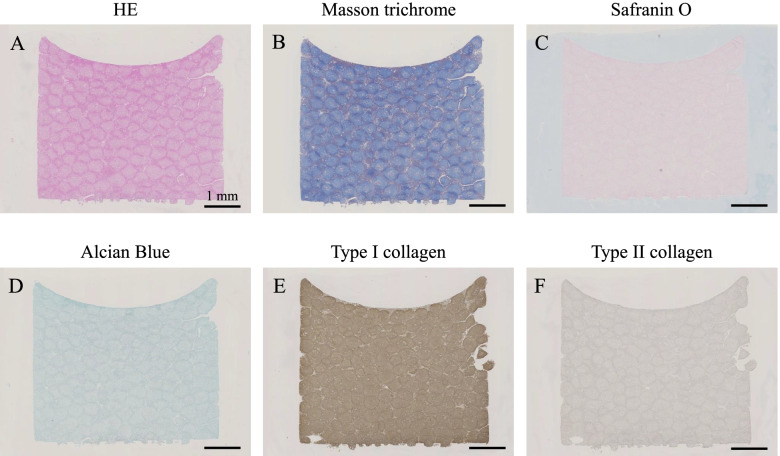
Histological characteristics of the three-dimensional construct. The sections were stained with haematoxylin & eosin (HE), Masson’s trichrome, Safranin O, and Alcian blue, and immunohistochemistry staining of type I and type II collagen. Scale bar = 1 mm

The boundaries of the spheroids were clear but tended to fuse with each other in HE sections (Fig. 5a). Numerous cell nuclei were confirmed inside and around the spheroids, and ECM production was observed in the section stained by Masson’s trichrome (Fig. 5b). The sections stained by Safranin O and Alcian blue were negative (Figs. 5c and d), but the expression of type I collagen was positively confirmed in the sections showing immunohistochemistry findings for type I collagen (Fig. 5e). However, immunohistochemical staining of type II collagen did not show positive findings (Fig. 5f).

### CT and MR assessments of the osteochondral defects

**Fig. 6:**
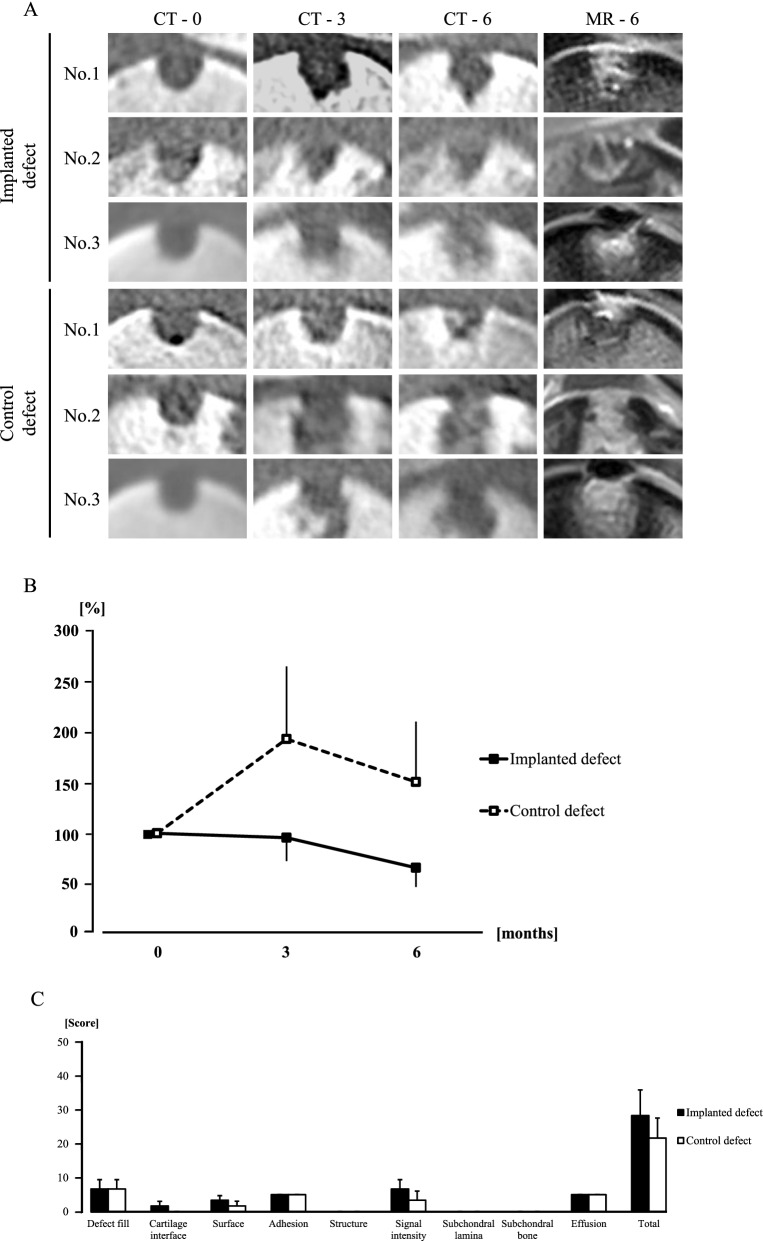
Computed tomography (CT) and magnetic resonance (MR) assessment of osteochondral defects. CT images show one cross-section of the multi-planar reconstruction images at one (CT-0), three (CT-3), and six (CT-6) months after the surgery in No. 1, No. 2, and No. 3, and MR images (MR-6) show the images corresponding to the CT images at 6 months after surgery (**A**). Line graph shows the averages of RV (radiolucent volume) percentages at the third and sixth months against those at month zero in both defects (**B**). Bar graph shows the averages of the items in the Modified 2D-MOCART scores based on the images of MR-6 (**C**)

The radiopaque area changed minimally 6 months after implantation in No. 1, and the area emerged from the boundary between the bone and the implant in the defects in No. 2 (Fig. 6a). However, the radiolucent area increased in size after implantation in No. 3 (Fig. 6a). The radiopaque area increased more steadily upward and inward in the control site in No. 1, but No. 2 and No. 3 showed large radiolucent areas in the control sites (Fig. 6a). The average RV values in both defects decreased at the third month and at the sixth month after the surgery, compared with those obtained immediately after surgery (at month 0) (Fig. 6b). The RV values and RV percentages relative to the values obtained at month 0 for each pony are summarized in Table 2. Numerical values were shown as mean ± standard error.

**Table 2:** RV value (mm^2^) and RV percentage (%) relative to the value at month 0

After surgery (months)	RV	No. 1		No. 2		No. 3		Mean ± SE	
		R	L	R	L	R	L	R	L
0	mm^2^	15.6	16.2	16.3	13.7	16.9	16.8	16.3 ± 0.4	15.5 ± 1.0
	%	100	100	100	100	100	100	100	100
3	mm^2^	15.3	12.3	8.8	44.1	22.7	30.3	15.6 ± 4.0	28.9 ± 9.2
	%	97.9	75.7	54.6	323.3	134.7	180.9	95.7 ± 23.2	193.3 ± 71.7
6	mm^2^	11.1	5.3	5.0	30.8	16.2	32.6	10.8 ± 3.2	22.9 ± 8.8
	%	71.2	32.7	30.7	225.3	95.9	194.5	65.9 ± 19.0	150.8 ± 59.7

RV, radiolucent volume; R, Right hindlimb; L, left hindlimb

MR images of the implanted sites of No. 1 showed that the signal pattern at the surface of the articular cartilage was almost restored to the normal signal intensity of the surrounding normal cartilage, whereas the formation of new bone under the cartilaginous tissue was incomplete (Fig. 6a). The high signal intensity indicated that synovial fluid had flowed into the subchondral area in the implanted sites, especially in Nos. 1 and 3. For No. 2, the defect was occupied by a fibrous tissue with medium signal intensity (Fig. 6a). On the other hand, a high signal-intensity area occupied the enlarged osteochondral defect at the control sites of all animals excluding No. 1 (Fig. 6a). There were differences in the outcome measures of the modified 2D-MOCART grading scale such as the cartilage interface, surface, signal intensity, and total scores (Fig. 6c). On the other hand, no difference was observed in the measures of defect fill adhesion, structure subchondral lamina, subchondral bone and effusion (Fig. 6c). The modified-2D MOCART scores for each pony are summarized in Table 3. Numerical values were shown as mean ± standard error.

**Table 3:** Modified-2D MOCART score

Feature		Score	No. 1		No. 2		No. 3		Mean ± SE	
			R	L	R	L	R	L	R	L
Defect fill	Complete	20	10	10	10	10	0	0	6.7 ± 2.7	6.7 ± 2.7
	Hypertrophy	15								
	Incomplete > 50%	10								
	Incomplete > 50%	5								
	Subchondral bone expose	0								
Cartilage interface	Complete	15	0	0	5	0	0	0	1.7 ± 1.4	0.0 ± 0.0
	Demarcating border visible	10								
	Defect visible < 50%	5								
	Defect visible > 50%	0								
Surface	Intact	10	5	5	5	0	0	0	3.3 ± 1.4	1.7 ± 1.4
	Damaged < 50% of depth	5								
	Damaged > 50% of depth	0								
Adhesion	No	5	5	5	5	5	5	5	5.0 ± 0.0	5.0 ± 0.0
	Yes	0								
Structure	Homogenous	5	0	0	0	0	0	0	0.0 ± 0.0	0.0 ± 0.0
	Inhomogenous / cleft formation	0								
Signal intensity	Normal	30	10	10	10	0	0	0	6.7 ± 2.7	3.3 ± 2.7
	Nearly normal	10								
	Abnormal	0								
Subchondral lamina	Intact	5	0	0	0	0	0	0	0.0 ± 0.0	0.0 ± 0.0
	Not intact	0								
Subchondral bone	Intact	5	0	0	0	0	0	0	0.0 ± 0.0	0.0 ± 0.0
	Granulation tissue/ cyst / sclerosis	0								
Effusion	No effusion	5	5	5	5	5	5	5	5.0 ± 0.0	5.0 ± 0.0
	Effusion	0								
Total (0–100)			35	35	40	20	10	10	28.3 ± 7.6	21.7 ± 5.9

R, Right hindlimb; L, left hindlimb; MOCART, magnetic resonance observation of cartilage repair tissue

### Arthroscopic (macroscopic) assessment of the osteochondral defects

**Fig. 7:**
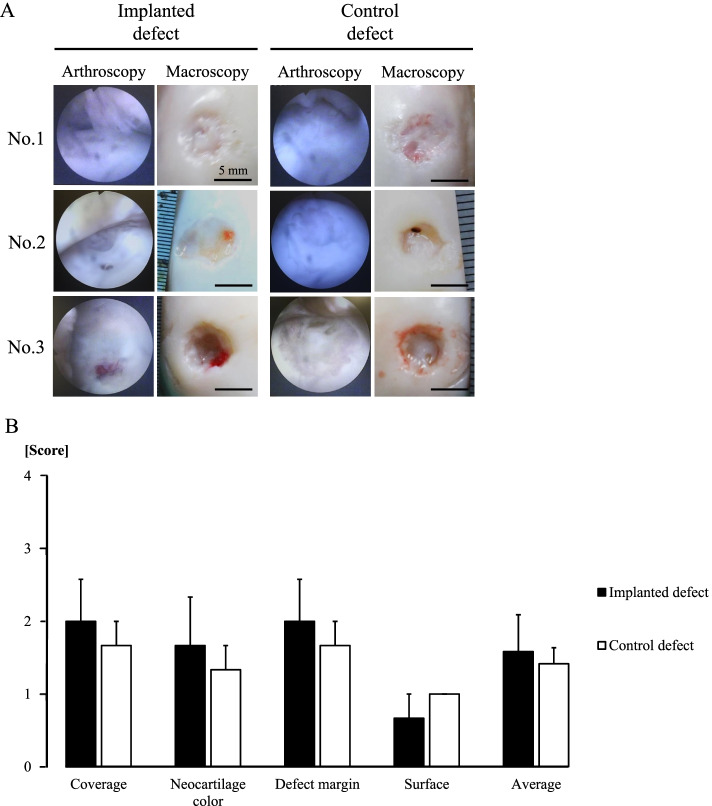
Arthroscopic (macroscopic) assessment of osteochondral defects. Arthroscopic and macroscopic findings of the surface are shown. Scale bar = 5 mm (**A**). Macroscopic scores were evaluated with ICRS gross grading scale based on the macroscopic findings (**B**)

Gross pathology in the implanted sites of Nos. 1 and 2 and the control site of No. 1 showed a smooth surface compared to the implanted site of No. 3 and the control sites of Nos. 2 and 3 (Fig. 7a). There were differences in all measures such as coverage, neocartilage colour, defect margin, surface, and average of all scores (Fig. 7b). The scores in the ICRS gross grading scale for each pony are summarized in Table 4. Numerical values are shown as mean ± standard error.

**Table 4:** ICRS gross grading scale

Feature		Score	No. 1		No. 2		No. 3		Mean ± SE	
			R	L	R	L	R	L	R	L
Coverage	> 75% fill	4	3	2	2	2	1	1	2.0 ± 0.6	1.7 ± 0.3
	50–75% fill	3								
	25–50% fill	2								
	< 25% full	1								
	No fill	0								
Neocartilage colour	Normal	4	3	2	1	1	1	1	1.7 ± 0.7	1.3 ± 0.3
	25% yellow/brown	3								
	50% yellow/brown	2								
	75% yellow/brown	1								
	100%yellow/brown	0								
Defect margins	Invisible	4	3	2	2	2	1	1	2.0 ± 0.6	1.7 ± 0.3
	25% circumference visible	3								
	50% circumference visible	2								
	75% circumference visible	1								
	Entire circumference visible	0								
Surface	Smooth/level with normal	4	1	1	1	1	0	1	0.7 ± 0.3	1.0 ± 0.0
	Smooth but raised	3								
	Irregular 25–50%	2								
	Irregular 50–75%	1								
	Irregular > 75%	0								
Average (0–4)			2.5	1.8	1.5	1.5	0.8	1.0	1.6 ± 0.5	1.4 ± 0.2

R, Right hindlimb; L, left hindlimb; ICRS, International Cartilage Repair Society

### Histological assessment of the osteochondral defects

**Fig. 8:**
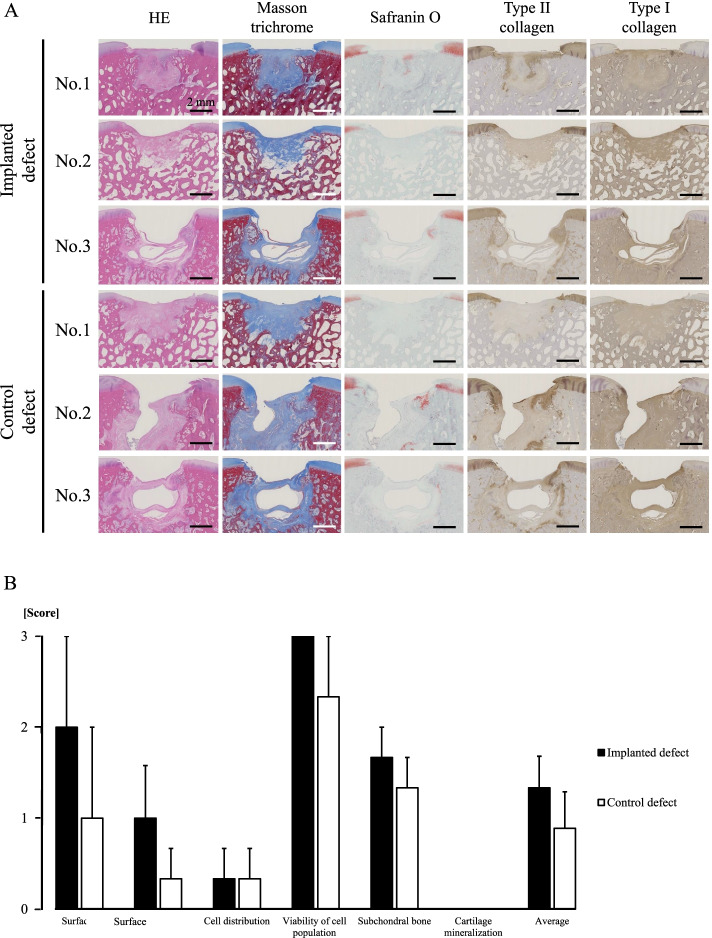
Histological assessment of osteochondral defects. The sections were stained using haematoxylin & eosin (HE), Masson’s trichrome, Safranin O, and immunohistochemical staining of type I and type II collagen. Scale bar = 2 mm (**A**). Microscopic scores were calculated with the ICRS histological grading scale based on histological findings (**B**)

Histopathological findings in No. 1 indicated smooth coverage containing cartilage matrix positively stained by Safranin O and immunohistochemistry of type II collagen as well as subchondral bone formation in the implanted sites (Fig. 8a), while a deeply recessed surface and a lower rate of bone formation were observed in the control defects of Nos. 2 and 3 (Fig. 8a). There were differences in the ICRS histologic scores, except for cell distribution and cartilage mineralization between the implanted sites and the control sites (Fig. 8b). Each score in ICRS histological grading scale for each pony is summarized in Table 5. Numerical values are shown as mean ± standard error.

**Table 5:** ICRS histological grading scale scores

Feature		Score	Horse No. 1		Horse No. 2		Horse No. 3		Mean ± SE	
			R	L	R	L	R	L	R	L
Surface	Smooth/continuous	3	3	3	3	0	0	0	2.0 ± 1.0	1.0 ± 1.0
	Discontinuities/irregulatrity	0								
Matrix	Hyaline	3	2	1	1	0	0	0	1.0 ± 0.6	0.3 ± 0.3
	Mixture; hyaline/fibrocartilage	2								
	Fibrocartilage	1								
	Fibrous tissue	0								
Cell distribution	Columnar	3	1	1	0	0	0	0	0.3 ± 0.3	0.3 ± 0.3
	Mixed/columnar clusters	2								
	Clusters	1								
	Individual cells/disorganized	0								
Viability of cell population	Predominantly viable	3	3	3	3	1	3	3	3.0 ± 0.0	2.3 ± 0.7
	Partially viable	1								
	< 10% viable	0								
Subchondral bone	Normal	3	2	2	2	1	1	1	1.7 ± 0.3	1.3 ± 0.3
	Increased remodeling	2								
	Bone necrosis/granulation tissue	1								
	Detached/fracture/callus at base	0								
Cartilage mineralization (calcified cartilage)	Normal	3	0-	0	0	0	0	0	0.0 ± 0.0	0.0 ± 0.0
	Abnormal/inappropriate location	0								
Average (0–3)			1.8	1.7	1.5	0.33	0.7	0.7	1.3 ± 0.4	0.9 ± 0.4

RF, Right forelimb; LF, left forelimb; ICRS, International Cartilage Repair Society

## Discussion

In this study, we succeeded in producing a cylindrical 3D construct by placing equine SM-MSCs aggregates into a cylindrical mould. We also attempted to regenerate the articular cartilage and subchondral bone by autologously implanting the construct into the osteochondral defects created at the loading-bearing site of the medial femoral condyle in ponies.

SM-MSCs isolated from the carpal joint of the ponies were cultured using a mixed medium with low-serum and serum-free human MSC culture media. Based on the measurements of cell doubling number and time, the cultured SM-MSCs were confirmed to grow much faster than those described in previous reports [17, 18]. In this study, the cell culture was performed only using the mixed medium; unlike previous studies, we did not use Minimum Essential Medium or Dulbecco’s Modified Eagle Medium containing 10% FBS [17, 18]. Moreover, cell aging analysis was not performed in this study. Therefore, it is necessary to investigate the effect of the medium on cell senescence in the next study.

The results of flow cytometry for surface antigens of the obtained cells showed almost the same results as those previously reported for equine SM-MSCs [19, 20]. However, some differences were observed for CD105 and MHC class II. Future studies should also attempt to investigate the factors that influence the population of CD90-positive cells in SM-MSCs, such as the medium or passage number.

The cells used in the tri-linage differentiation were cultured using the mixed medium as described above, and we confirmed that they possessed multipotency at P3. However, it may be necessary to investigate the retention of multipotency (especially chondrogenic and osteogenic differentiation ability) on each passage.

The histological assessments of the constructs consisting of the cultured SM-MSCs showed positive results for type I collagen and negative results for type II collagen, safranin, and Alcian blue. Thus, we confirmed that the construct did not have cartilaginous characteristics before implantation. Moreover, the boundaries between spheroids were visible; thus, their fusion was considered to be inadequate. Although the mechanical strength of the constructs was not examined in this study, the constructs appeared to be barely graspable by tweezers and may not have had sufficient strength to be examined in the mechanical strength test. In this study, the medium used for cell culture was also used for construct preparation. However, more detailed examinations using different media and culture periods, as reported previously [21], may yield a construct with stronger mechanical properties.

A radiopaque area emerged from the boundary between the implant and the bone defect, and RVs at the implanted defects decreased 3 and 6 months after surgery compared with those recorded immediately after surgery. On the other hand, the control defects grew in size after the surgery. Experimentally created osteochondral defects on the loading surface of the medial femoral condyle have been shown to progressively expand 3 weeks after surgery [22]. In addition, enlargement of osteochondral defects at femoral medial condyles has been reported to be caused by autologous transplantation of cancellous bone [23]. Furthermore, in a recent study using finite element analysis, osteochondral defects were revealed to have expanded owing to the tension applied in the cranio-caudal direction around the defect in the developing subchondral bone cyst [24]. Thus, the artificial osteochondral defect in this study may have enlarged as a result of the tension generated around the defect, and the implantation of the cell construct would have facilitated osteochondral regeneration and prevented the enlargement of the defect.

By performing MR assessments in addition to the CT assessment, it became possible to evaluate not only the subchondral bone formation but also the cartilage regeneration. Therefore, we were able to obtain findings indicating the possibility of cartilage regeneration at the implanted site of No. 1. However, MR assessment was performed using 2D images in this study and was not conducted by directly measuring the volume of cartilage tissue as in the CT assessments. Recently, software for measuring the volume of cartilage tissue using an MR device has been developed [25, 26]. Accordingly, it will be important to evaluate the volume of regenerated cartilage by using such software.

In this study, macroscopic findings at autopsy were evaluated based on the ICRS score, which included arthroscopic assessments performed before autopsy. In No. 1, gross pathology of the implanted sites showed higher scores for various aspects, including coverage, neocartilage colour, and defect margins, than that for the control sites. However, in Nos. 2, and 3, there were no differences between the implanted and the control sites. As speculated, the absence of differences in macroscopic scores could be attributed to the absence of cartilage regeneration in these two animals. However, this result can be inferred from the results of the MR assessment. Moreover, using the 3D MR analysis as previously reported [25, 26], more detailed cartilage evaluation can be performed non-invasively.

Histological findings obtained 6 months after implantation showed that cartilage regeneration was particularly confirmed in No. 1, and bone regeneration was particularly observed in No. 2. Although cartilage regeneration was observed at the implanted site in No. 1, subchondral bone reconstruction at the implanted site was inferior to that of the control site. Because bone tissue is naturally repaired, it is quite possible that the subchondral bone was remodelled similar to the control site in No. 1. However, since hyaline cartilage has extremely low self-repairing ability, cartilage regeneration was promoted at the implanted site in No. 1, as previously reported [27]. In No. 2, the osteochondral defect was significantly enlarged at the control site, while the defect at the implanted site was reduced owing to the differentiation of the construct into bone [27]. The mechanism of regeneration mainly involved the migration of MSCs to the injured area and differentiation according to the surrounding tissue, as well as the release of various types of growth factors and cytokines that are supposed to promote tissue repair [28–30]. For the implanted site in No. 3, bone regeneration was confirmed without cartilage regeneration, as observed in No. 2.

As the osteochondral defects were created on the femoral medial condyle for this study, the constructs were implanted in the direction that would be against gravity when the pony woke and stood up after surgery. Subsequently, since the ponies were allowed to stand and walk freely in this study, the constructs could fall off the defects after implantation owing to gravity.

## Conclusions

In this study, scaffold-free 3D constructs consisting of only equine SM-MSCs were created using a mould, and articular cartilage and subchondral bone were regenerated on implanting the constructs. The results confirmed that implantation of these constructs can promote regeneration of cartilage and bone in the equine knee joint. Those findings may lead to the development of new treatments for subchondral bone cysts.

## Data Availability

All data generated or analysed during this study are included in this article.

## Abbreviations

3D: three-dimensional
CT: computed tomography
DMEM: Dulbecco’s modified Eagle medium
FITC: fluorescein isothiocyanate
HE: haematoxylin & eosin
ICRS: International Cartilage Repair Society
MFI: mean fluorescence intensity
MHC: major histocompatibility complex
MOCART: magnetic resonance observation of cartilage repair tissue
MR: magnetic resonance
MSCs: mesenchymal stem cells
OA: osteoarthritis
P2: passage 2
PBS: phosphate-buffered saline
PCM: primary culture medium
RV: radiolucent volume
SB: staining buffer
SCM: secondary culture medium
SF: synovial fluid
SF-MSCs: SF-derived MSCs
SM-MSCs: synovial membrane-derived mesenchymal stem cells
